# Supplementary material for: Non-obese non-alcoholic fatty liver disease and the risk of chronic kidney disease: a systematic review and meta-analysis
Source: PeerJ. 2024 Dec 17;12:e18459. doi: 10.7717/peerj.18459 (PMC11660860; doi:10.7717/peerj.18459)
Supplement: Supplemental Information 2 [file peerj-12-18459-s002.docx]

**Supplementary Table 1**

**Full search strategies for “Non-obese nonalcoholic fatty liver disease and the risk of incident chronic kidney disease: A literature review and meta-analysis”**

**Pubmed**

#1

**MeSH**

Non-alcoholic Fatty Liver Disease

**Entry Terms**

Non alcoholic Fatty Liver Disease

NAFLD

Nonalcoholic Fatty Liver Disease

Fatty Liver, Nonalcoholic

Fatty Livers, Nonalcoholic

Liver, Nonalcoholic Fatty

Livers, Nonalcoholic Fatty

Nonalcoholic Fatty Liver

Nonalcoholic Fatty Livers

Nonalcoholic Steatohepatitis

Nonalcoholic Steatohepatitides

Steatohepatitides, Nonalcoholic

Steatohepatitis, Nonalcoholic

NASH

**Search strategy**: ("Non-alcoholic Fatty Liver Disease"[Mesh]) OR ((((((((((((((Non alcoholic Fatty Liver Disease[Title/Abstract]) OR (NAFLD[Title/Abstract])) OR (Nonalcoholic Fatty Liver Disease[Title/Abstract])) OR (Fatty Liver, Nonalcoholic[Title/Abstract])) OR (Fatty Livers, Nonalcoholic[Title/Abstract])) OR (Liver, Nonalcoholic Fatty[Title/Abstract])) OR (Livers, Nonalcoholic Fatty[Title/Abstract])) OR (Nonalcoholic Fatty Liver[Title/Abstract])) OR (Nonalcoholic Fatty Livers[Title/Abstract])) OR (Nonalcoholic Steatohepatitis[Title/Abstract])) OR (Nonalcoholic Steatohepatitides[Title/Abstract])) OR (Steatohepatitides, Nonalcoholic[Title/Abstract])) OR (Steatohepatitis, Nonalcoholic[Title/Abstract])) OR (NASH[Title/Abstract]))

#2

**MeSH**

Thinness

**Entry Terms**

Leanness

Underweight

Lean

Thin

Non obese

Nonobesity

Non obesity

non obese

normal weight

non overweight

nonoverweight

non obesity

**Search strategy:** (((((((((((Leanness[Title/Abstract]) OR (Underweight[Title/Abstract])) OR (lean[Title/Abstract])) OR (non-obese[Title/Abstract])) OR (non obese[Title/Abstract])) OR (nonoverweight[Title/Abstract])) OR (non overweight[Title/Abstract])) OR (normal weight[Title/Abstract])) OR (thin[Title/Abstract])) OR (nonobesity[Title/Abstract])) OR (non obesity[Title/Abstract])) OR ("Thinness"[Mesh])

#3

**MeSH**

Renal Insufficiency, Chronic

**Entry Terms:**

Chronic Renal Insufficiencies

Renal Insufficiencies, Chronic

Chronic Renal Insufficiency

Kidney Insufficiency, Chronic

Chronic Kidney Insufficiency

Chronic Kidney Insufficiencies

Kidney Insufficiencies, Chronic

Chronic Kidney Diseases

Chronic Kidney Disease

Disease, Chronic Kidney

Diseases, Chronic Kidney

Kidney Disease, Chronic

Kidney Diseases, Chronic

Chronic Renal Diseases

Chronic Renal Disease

Disease, Chronic Renal

Diseases, Chronic Renal

Renal Disease, Chronic

Renal Diseases, Chronic

CKD

kidney function

kidney failure

renal disease

glomerular filtration rate

GFR

estimated glomerular filtration rate

eGFR

creatinine

albuminuria

microalbuminuria

macroalbuminuria

proteinuria

kidney injury

**Search stratery:** (((((((((((((((((((((((((((((((((Chronic Renal Insufficiencies[Title/Abstract]) OR (Renal Insufficiencies, Chronic[Title/Abstract])) OR (Chronic Renal Insufficiency[Title/Abstract])) OR (Kidney Insufficiency, Chronic[Title/Abstract])) OR (Chronic Kidney Insufficiency[Title/Abstract])) OR (Chronic Kidney Insufficiencies[Title/Abstract])) OR (Kidney Insufficiencies, Chronic[Title/Abstract])) OR (Chronic Kidney Diseases[Title/Abstract])) OR (Chronic Kidney Disease[Title/Abstract])) OR (Disease, Chronic Kidney[Title/Abstract])) OR (Diseases, Chronic Kidney[Title/Abstract])) OR (Kidney Disease, Chronic[Title/Abstract])) OR (Kidney Diseases, Chronic[Title/Abstract])) OR (Chronic Renal Diseases[Title/Abstract])) OR (Chronic Renal Disease[Title/Abstract])) OR (Disease, Chronic Renal[Title/Abstract])) OR (Diseases, Chronic Renal[Title/Abstract])) OR (Renal Disease, Chronic[Title/Abstract])) OR (Renal Diseases, Chronic[Title/Abstract])) OR (CKD[Title/Abstract])) OR (kidney function[Title/Abstract])) OR (kidney failure[Title/Abstract])) OR (renal disease[Title/Abstract])) OR (glomerular filtration rate[Title/Abstract])) OR (GFR[Title/Abstract])) OR (eGFR[Title/Abstract])) OR (estimated glomerular filtration rate[Title/Abstract])) OR (creatinine[Title/Abstract])) OR (albuminuria[Title/Abstract])) OR (microalbuminuria[Title/Abstract])) OR (macroalbuminuria[Title/Abstract])) OR (proteinuria[Title/Abstract])) OR (kidney injury[Title/Abstract])) OR ("Renal Insufficiency, Chronic"[Mesh])

1# AND 2# AND 3#

**Web of science**

#1

((((((((((((((TS=(Non-alcoholic Fatty Liver Disease)) OR TS=(Non alcoholic Fatty Liver Disease)) OR TS=(NAFLD)) OR TS=(Nonalcoholic Fatty Liver Disease)) OR TS=(Fatty Liver, Nonalcoholic)) OR TS=(Fatty Livers, Nonalcoholic)) OR TS=(Liver, Nonalcoholic Fatty)) OR TS=(Livers, Nonalcoholic Fatty)) OR TS=(Nonalcoholic Fatty Liver)) OR TS=(Nonalcoholic Fatty Livers)) OR TS=(Nonalcoholic Steatohepatitis)) OR TS=(Nonalcoholic Steatohepatitides)) OR TS=(Steatohepatitides, Nonalcoholic)) OR TS=(Steatohepatitis, Nonalcoholic)) OR TS=(NASH)

#2

((((((((((((TS=(Leanness)) OR TS=(Underweight)) OR TS=(Thinness)) OR TS=( lean)) OR TS=(thin)) OR TS=(non-obese)) OR TS=(underweight)) OR TS=(nonobesity)) OR TS=(non obese)) OR TS=(normal weight)) OR TS=(non overweight)) OR TS=(nonoverweight)) OR TS=(non obesity)

#3

((((((((((((((((((((((((((((((((TS=(Chronic Renal Insufficiencies)) OR TS=(Renal Insufficiencies, Chronic)) OR TS=(Chronic Renal Insufficiency)) OR TS=(Kidney Insufficiency, Chronic)) OR TS=(Chronic Kidney Insufficiency)) OR TS=(Chronic Kidney Insufficiencies)) OR TS=(Kidney Insufficiencies, Chronic)) OR TS=(Chronic Kidney Diseases)) OR TS=(Chronic Kidney Disease)) OR TS=(Disease, Chronic Kidney)) OR TS=(Diseases, Chronic Kidney)) OR TS=(Kidney Disease, Chronic)) OR TS=(Kidney Diseases, Chronic)) OR TS=(Chronic Renal Diseases)) OR TS=(Chronic Renal Disease)) OR TS=(Disease, Chronic Renal)) OR TS=(Diseases, Chronic Renal)) OR TS=(Renal Disease, Chronic)) OR TS=(Renal Diseases, Chronic)) OR TS=(CKD)) OR TS=(kidney function)) OR TS=(kidney failure)) OR TS=(renal disease)) OR TS=(glomerular filtration rate)) OR TS=(GFR)) OR TS=(estimated glomerular filtration rate)) OR TS=(eGFR)) OR TS=(creatinine)) OR TS=(albuminuria)) OR TS=(microalbuminuria)) OR TS=(macroalbuminuria)) OR TS=(proteinuria)) OR TS=(kidney injury)

1# AND 2# AND 3#

**EMBASE**

#44 #41 AND #42 AND #43

#43 #25 OR #26 OR #27 OR #28 OR #29 OR #30 OR #31 OR #32 OR #33

#42 #8 OR #36 OR #37

#41 #7 OR #40

#40 #38 OR #39

#39 'nash (nonalcoholic steatohepatitis)' OR 'non alcohol steato-hepatitis' OR 'non alcohol steatohepatitis' OR 'non alcoholic steato-hepatitis' OR 'non-alcohol steato-hepatitis' OR 'non-alcohol steatohepatitis' OR 'non-alcoholic steatohepatitis' OR 'non-alcoholic steatosis hepatitis' OR 'non-alcoholic steatotic hepatitis' OR 'nonalcohol steato-hepatitis' OR 'nonalcohol steatohepatitis' OR 'nonalcoholic fatty liver inflammation' OR 'nonalcoholic steato-hepatitis' OR 'nonalcoholic steatosis hepatitis' OR 'nonalcoholic steatotic hepatitis' OR 'nonalcoholic steatohepatitis'nafld2023-07-162023-07-1626360

#38'nonalcoholic steatohepatitis'/exp

#37 #34 OR #35

#36'leanness'/exp

#35'body lean mass' OR 'body mass, lean' OR 'body weight, lean' OR 'lean body mass' OR 'lean weight' OR 'weight, lean' OR 'lean body weight'

#34 'lean body weight'/exp

#33 #5 OR #6

#32 #23 OR #24

#31 #21 OR #22

#30 #19 OR #20

#29 #17 OR #18

#28 #15 AND #16

#27 #13 OR #14

#26 #11 OR #12

#25 #9 OR #10

#24 'acute renal injury' OR 'chronic kidney injury' OR 'chronic renal injury' OR 'kidney cortex lesion' OR 'kidney damage' OR 'kidney lesion' OR 'kidney trauma' OR 'renal damage' OR 'renal injury' OR 'renal lesion' OR 'renal trauma' OR 'trauma, kidney' OR 'trauma, renal' OR 'kidney injury'

#23 'kidney injury'/exp

#22 'digestive proteinuria' OR 'tubule proteinuria' OR 'proteinuria'

#21 'proteinuria'/exp

#20 'macro-albuminuria' OR 'macroalbuminuria'

#19 'macroalbuminuria'/exp

#18 'albumin creatinine clearance ratio' OR 'albuminuria, micro' OR 'micro-albuminuria' OR 'pauci albuminuria' OR 'relative albumin clearance' OR 'microalbuminuria'

#17 'microalbuminuria'/exp

#16 'albuminuria'

#15 'albuminuria'/exp

#14 '1 methylglycocyamidine' OR '1 methylhydantoin 1 imide' OR '2 imino 1 methyl 4 imidazolinone' OR 'creatinin' OR 'creatinine hydrochloride' OR 'kreatinine' OR 'methylglycocyamimine' OR 'creatinine'

#13 'creatinine'/expn

#12 'egfr (estimated glomerular filtration rate)' OR 'estimated gfr' OR 'estimated glomerulofiltration rate' OR 'estimated glomerulus filtration rate' OR 'estimated glomerular filtration rate'

#11 'estimated glomerular filtration rate'/exp

#10 'gfr (glomerulus filtration rate)' OR 'glomerular filtration rate' OR 'glomerulofiltration rate' OR 'kidney gfr' OR 'kidney glomerulus filtration rate' OR 'glomerulus filtration rate'

#9 'glomerulus filtration rate'/exp

#8 #3 OR #4

#7 #1 OR #2

#6 'chronic kidney disease' OR 'chronic kidney disorder' OR 'chronic kidney insufficiency' OR 'chronic nephropathy' OR 'chronic renal disease' OR 'chronic renal failure' OR 'chronic renal insufficiency' OR 'kidney chronic failure' OR 'kidney disease, chronic' OR 'kidney failure, chronic' OR 'kidney function, chronic disease' OR 'renal insufficiency, chronic' OR 'chronic kidney failure'

#5 'chronic kidney failure'/exp

#4 'thinness' OR 'weight insufficiency' OR 'underweight'

#3 'underweight'/exp

#2 'nafld (nonalcoholic fatty liver disease)' OR 'non alcoholic fatty liver disease' OR 'non alcoholic hepato-steatosis' OR 'non alcoholic hepatosteatosis' OR 'non alcoholic liver steatosis' OR 'non alcoholic steatotic hepatopathy' OR 'non-alcoholic fatty liver' OR 'non-alcoholic fatty liver disease' OR 'non-alcoholic fld' OR 'non-alcoholic hepatic steatosis' OR 'nonalcoholic fatty liver disease' OR 'nonalcoholic fld' OR 'nonalcoholic hepatic steatosis' OR 'nonalcoholic hepatosteatosis' OR 'nonalcoholic liver steatosis' OR 'nonalcoholic fatty liver'

#1 'nonalcoholic fatty liver'/exp
