# Supplementary material for: Non-obese non-alcoholic fatty liver disease and the risk of chronic kidney disease: a systematic review and meta-analysis
Source: PeerJ. 2024 Dec 17;12:e18459. doi: 10.7717/peerj.18459 (PMC11660860; doi:10.7717/peerj.18459)
Supplement: Supplemental Information 3 [file peerj-12-18459-s003.docx]

**Table 1 Characteristics of included studies.**

| First author | Country | Study design | Non-lean NAFLD/lean NAFLD | Exposure Disease and Definition | Outcome Disease and Definition | Exclusion | Score |
| --- | --- | --- | --- | --- | --- | --- | --- |
| Akahane et al. 2020 | Japan | cross-sectional study | 590/507 | Nonalcoholic fatty liver disease (NAFLD). **Definition**: Fatty liver was defined by ultrasonography when there was hepatic parenchymal brightness and liver-to-kidney contrast  Obesity: **Definition** Obesity was defined as a BMI ≥25 kg/m2 | CKD. **Definition**: CKD was defined as an estimated GFR <60 mL/min/1.73 m2 **Note:** estimated glomerular filtration rate (eGFR) (mL/min/1.73 m2) = 194 × (serum creatinine level (mg/dL)) − 1.094 × (age (y)) − 0.287. The product of this equation was multiplied by a correction factor of 0.739 for women. | 1) Individuals with hepatitis B surface antigen- or anti-hepatitis C virus antibody-positive or consumed alcohol (men and women: >30 and >20 g/day of alcohol, respectively); 2) Adjusted for age, sex, and the Brinkman Index | 8 |
| Ampuero et al. 2018 | Spain | cross-sectional study | 713/345 | NAFLD.  **Definition**: NAFLD by liver biopsy  Obesity:  **Definition**: A BMI of 30 was used to distinguish obese from non‐obese patients. | CKD **Definition**: Chronic kidney disease was defined as eGFR ≤ 60 mL/min/1.73 m^2^. **Note**: estimate glomerular filtration rate (eGFR) using the following equation: GFR = 141 × min (Scr/κ,1)^α^ × max(Scr/κ,1)^−1.209^ × 0.993^Age^ × 1.018 [if female] × 1.159 [if black]. Scr is serum creatinine (mg/dL), κ is 0.7 for females and 0.9 for males, α is −0.329 for females and −0.411 for males, min indicates the minimum of Scr/κ or 1, and max indicates the maximum of Scr/κ or 1. | 1) Patients with significant alcohol intake (>20 g daily for men and >10 g daily for women) and evidence of viral or autoimmune hepatitis, HIV, drug‐induced fatty liver, or other metabolic liver diseases (such as haemochromatosis or Wilson's disease). | 7 |
| Chon et al. 2020 | Korea | prospective cohort study | 1140/459 | NAFLD. **Definition**: The presence of NAFLD was defined as a NAFLD liver fat score of ≥ –0.64031 and FIB-4 score was calculated in the NAFLD patients.  Obesity.  **Definition**: Participants were categorized as non-obese (BMI < 25 kg/m2) and obese (BMI ≥ 25 kg/m2) | CKD. **Definition**: CKD were defined as an eGFR of <60 mL/min/1.73m2 and/or proteinuria of more than 1+ on the dipstick.  Note: estimated GFR = 175 × standardized Scr^−1.154^ × age^−0.203^ × 1.212 [if black] × 0.742 [if female], where GFR is expressed as mL/min/1.73 m2 of body surface area41 and Scr is expressed in mg/dL. | 1. excessive alcohol consumption(>210g/wk for men, 140g/wk for women; 2)hepatitis; 3) CKD at baseline ;4) missing data for eGFR and fatty score; 5) missing data for follow-up body weight and waist-to-hip ratio; 6) lost to follow-up; 7) malignancy | 7 |
| Fan et al. 2023 | UK Biobank | prospective cohort | 100464/2160 | NAFLD. **Definition:** Individuals who with fatty liver but without excess alcohol intake were defined as NAFLD. Note: fatty liver index (FLI). Individuals with FLIs ≥60 were defined as fatty liver cases. Alcohol intake ≥30 g/day for men and ≥20 g/day for women were defined as excess alcohol intake.  Obesity.  **Definition**: Participants who had BMIs of 18.5–24.9, 25–29.9, or ≥30 kg/m^2^ were defined as normal weight, overweight, and obesity, respectively. | CKD. **Definition:** ICD-10 code. Incident diseases: I12.0, I13.1, N03.2–N03.7, N05.2–N05.7, N18.x, N19.x, N25.0, Z49.0–Z49.2, Z94.0, Z99.2.  Prevalent diseases: I21.x, I22.x, I25.2, I09.9, I11.0, I13.0, I13.2, I25.5, I42.0, I42.5–I42.9, I43.x, I50.x, P29.0. | 1) Participants who were missing data on alcohol consumption and FLI-related variates were excluded. | 6 |
| Hu et al. 2022 | China | cross-sectional study |  | MAFLD.  **Definition:**  MAFLD include evidence of hepatic steatosis (ultrasound) plus one of the following: evidence of no less than two of the following metabolic risk factors: (1) waist circumference ≥102/88 cm in Caucasian men and women (or ≥90/80cm in Asian men and women); (2) blood pressure ≥130/85mmHg or related drug treatment; (3) plasma triglycerides ≥150 mg/dl or related drug treatment; (4) plasma high density lipoprotein cholesterol (HDL-C) <40 mg/dl in men and <50 mg/dl in women or related drug treatment; (5) prediabetes (i.e. fasting glucose level of 100–125mg/dl, or 2-h post-load glucose level of 140–199mg/dl or HbA1c 5.7–6.4%); (6) homeostasis model assessment of insulin resistance (HOMA-IR) score ≥2.5; and (7) plasma high-sensitivity CRP level >2mg/L.  Obesity.  **Definition**: Overweight or obesity was defined as body mass index (BMI) ≥25kg/m2 in Caucasians or BMI ≥23kg/m2 in Asians. | CKD.  **Definition:** CKD was defined as an estimated glomerular filtration rate (eGFR) <60 mL/min/1.73 m^2^ (CKD-EPI) and/or the presence of albuminuria, defined as a urinary albumin-to-creatinine ratio (UACR) 30 mg/g. | 1) Other aetiologic factors known to be related to chronic liver disease (alcohol intake, >20 g/d; autoimmune hepatitis; and hepatitis B antigen or hepatitis C virus antibody positivity) were excluded; 2) Presence of history of liver or gallbladder surgery; 3) Hepatitis, gallbladder polyps, or inflammatory bowel disease. | 8 |
| Hu et al. 2022 | China | prospective cohort | 6117/4194 | NAFLD.  **Definition:**  Participants with FLD were defined as having NAFLD.  Obesity. **Definition**: BMI ≥ 25 according to the WHO’s proposed classification for Asian populations | CKD **Definition:**  CKD was characterized with eGFR < 60 mL/min/1.73 m^2^, in which eGFR was calculated as 141 x (SCr/0.9)^-0.411^ x (0.993)^age^ for men with SCr ≤ 0.9 mg/dL and 141 x (SCr/0.9)^-1.209^ x (0.993)^age^ for men with SCr > 0.9. | 1) those with alcohol intake missing or with excessive alcohol consumption (alcohol intake ≥ 30 g/d) according to the American Association for the Study of Liver Diseases Practice Guidance; 2) those with hepatitis B surface antigen positive or missing ; 3) those with a previous history of cardiovascular diseases (CVD, cancers , or liver cirrhosis or with an estimated glomerular filtration rate (eGFR) < 60 mL/min/1.73 m^2^ before FLD diagnosis, in which eGFR was estimated using the Chronic Kidney Disease Epidemiology Collaboration (CKD-EPI) study equation. | 7 |
| Iwaki et al 2022 | South Korea | retrospective cohort study | 165/58 | NAFLD.  **Definition:**  Biopsy-proven  Obesity.  **Definition**: We defined NAFLD with BMI <25 kg/m^2^ non obese NAFLD and NAFLD with BMI ≥25 kg/m^2^ as obese NAFLD. | NA | The exclusion criteria were other hepatic diseases, such as  hepatitis C, hepatitis B, autoimmune hepatitis, primary biliary cirrhosis, sclerosing cholangitis, Wilson’s disease, hemochromatosis, α1-antitrypsin deficiency, drug-induced steatohepatitis, and current or past history of significant alcohol consumption (>30 and >20 g/day of ethanol in men and women, respectively). Moreover, patients with coexisting malignancies and pregnant women were excluded. | 6 |
| Kim et al. 2022 | Korea | cross-sectional | 1274/525 | NAFLD. **Definition:** the comprehensive NAFLD score (CNS) and the NAFLD liver fat score (LFS); a CNS of ≥40 or an LFS of ≥ –0.640 were considered indicative of NAFLD.  Obesity.  **Definition**: Based on the criteria for the Asian-Pacific region,25 considered obese at BMI ≥25 kg/m. | NA | 1) those with age <40 years; 2) those with HBV, HCV infection, HCC or alcohol drink; 3) those with no data of BMI, WC, platelet count, AST, or ALT . | 6 |
| Kwon et al. 2023 | Korea | retrospective cohort | 786/4540 | NAFLD.  **Definition:** Abdominal ultrasound. NAFLD was defined when there was evidence of hepatic steatosis on ultrasonography without excessive alcohol intake or other concomitant liver diseases such as viral hepatitis. **Note**: 1) The presence of hepatic steatosis was assessed by abdominal ultrasonography using standard criteria such as parenchymal brightness, liver-to-kidney contrast, deep beam attenuation, and bright vessel walls; 2) Excessive alcohol intake was defined as > 20 g/day in men and > 10 g/day in women.  Obesity.  **Definition**: Overweight and obesity were defined as BMI ≥ 23 kg/m2 and ≥ 25 kg/m2, respectively, according to Asian-specific criteria. | CKD  **Definition:** CKD was defined as an eGFR less than 60 mL/min/1.73 m^2^ and/or urine albumin/creatinine ratio greater than or equal to 30 mg/g. | 1) excluded subjects with a history of malignant diseases or history of liver cirrhosis 2) subjects with missing laboratory data, anthropometric measurements, health questionnaires, or questionnaires regarding daily alcohol consumption. | 7 |
| Leung et al. 2017 | Hong Kong (China) | prospective cohort study | 235/72 | NAFLD. **Definition:** liver biopsy.  Obesity.  **Definition**: BMI less than 25kg/m^2^ was used to define the nonobese Asian population. The nonobese population was further divided into lean (<23kg/m^2^) and overweight (23-25 kg/ m^2^). | NA | 1) Patients with positive hepatitis B surface antigen or anti-hepatitis C virus (HCV) antibody with detectable HCV RNA, excessive alcohol consumption (≥20 g/day in men or ≥10 g/day in women), secondary fatty liver (e.g., use of systemic steroids or tamoxifen), or malignancies before baseline were excluded. | 6 |
| Liu et al. 2021 | UK Biobank (Caucasian ethnic) | prospective cohort | 49334/28468 | NAFLD.  **Definition:** Fatty liver index (FLI) for each participant and defined the hepatic steatosis as FLI ≥ 60. We defined < 30 g/day for men and < 20 g/day for women as low-to-moderate alcohol intake. NAFLD case was then defined as participants who with FLI ≥ 60 and had low-to-moderate alcohol drinking.  Obesity.  **Definition**: We also examined the effect of high BMI (i.e., BMI ≥ 30 kg/m^2^). | CKD.  **Definition:** ICD-10 code |  | 6 |
| Mikolasevic et al. 2020 | Croatia | Prospective study | 440/67 | NAFLD. **Definition:** Fibroscan-defined. Significant alcohol consumption was an exclusion criterion and it was defined as >30 g of alcohol per day (21 drinks per week) in men, and > 20 g/day (14 drinks per week) in women.  Obesity.  **Definition**: lean (BMI<25 kg/m2), overweight (BMI 25–29.9 kg/m2) or obese (BMI ≥ 30 kg/m2) | CKD **Definition:** Chronic kidney disease (CKD) was defined as presence of estimated glomerular filtration rate 60 mL/min/1.73 m^2^. | 1) Patients with viral, autoimmune or other metabolic chronic liver diseases were also excluded. Furthermore, we excluded from the analysis patients with malignancies, those who took drugs inducing hepatic steatosis (e.g. corticosteroids, high-dose estrogen, methotrexate or amiodarone), those with right-sided heart failure, and patients with missing data or transient elastography failure (including inadequate results). | 6 |
| Ragab et al. 2020 | Egypt | Prospective study | 27/14 | NAFLD. **Definition**: Abdominal ultrasonography.  Obesity.  **Definition**: | NA | 1) Patients with history of viral hepatitis, autoimmune hepatitis, or  other forms of chronic liver disease, those with self-reported acute infection within 2 weeks, and those with body mass index less than 18.0 kg/m^2^ were excluded from the study. | 6 |
| Yang et al. 2018 | China | retrospective cohort | 470/356 | NAFLD. **Definition**: ultrasonography. Obesity.  **Definition**: We defined obesity as a BMI of ≥25 kg/m^2^. | NA | 1) Subjects were excluded because of missing ultrasonography or blood biochemistry data, no evidence of fatty liver disease, heavy drinking (ethanol intake of≥140 g/week), serological positivity for hepatitis B or C, or being women with fatty liver disease. | 6 |
| Nabiet al. 2023 | France | Prospective study | 22089/3664 | NAFLD.  **Definition**: The fatty liver index (FLI). Subjects with FLI ≥60 were considered to have NAFLD.  Obesity.  **Definition**: Subjects were considered to be lean with a BMI <25kg/m², or <23 kg/m² if they were of Asian ethnicity and as overweight with a BMI 25-29.9 or 23-27.4 if they were of Asian ethnicity and obese with a BMI ≥30 kg/ ², ≥27.5 kg/m² if Asian ethnicity. | CKD. **Definition**: Chronic kidney disease was defined according to a glomerular filtration rate <60 ml/min/1.73m^2^. | 1) Subjects recruited for Constances were included in the present analysis if they were at least 18 years old, with no history of excess alcohol consumption defined by a daily consumption >30 g/day in men and >20 g/day in women, no history of chronic HBV or HCV infection, or history of other liver diseases except NAFLD. | 8 |
