# Supplementary material for: Non-obese non-alcoholic fatty liver disease and the risk of chronic kidney disease: a systematic review and meta-analysis
Source: PeerJ. 2024 Dec 17;12:e18459. doi: 10.7717/peerj.18459 (PMC11660860; doi:10.7717/peerj.18459)
Supplement: Supplemental Information 4 [file peerj-12-18459-s004.docx]

Rationale:

Nonalcoholic fatty liver disease (NAFLD) has emerged as the most common causes of chronic liver disease (CKD), impacting an estimated 25% of the population worldwide and 30% of population in China. The reciprocal relationships between NAFLD and obesity has been continuously reported. However, increasing data indicate that a proportion of subjects with NAFLD are lean or non-obese. The incidence of CKD in non-obese NAFLD patients compared with obese NAFLD remains unclear. Consistent conclusions regarding whether non-obese patients with NAFLD on the risk of developing CKD than obese patients are still not drawn.

Contribution:

To our knowledge, there are limited studies to assess the risk of CKD in non-obese NAFLD and obese NAFLD. Our study comprehensively assessed the risk of kidney damage in those two populations, and we not only assessed the risk of developing CKD, but also compared the eGFR, and serum creatinine, which reflected the kidney function. Our study will provide a deeper understanding of the relationship between NAFLD and CKD.
