# Supplementary figures and images for: Non-obese non-alcoholic fatty liver disease and the risk of chronic kidney disease: a systematic review and meta-analysis
Source: PeerJ. 2024 Dec 17;12:e18459. doi: 10.7717/peerj.18459 (PMC11660860; doi:10.7717/peerj.18459)

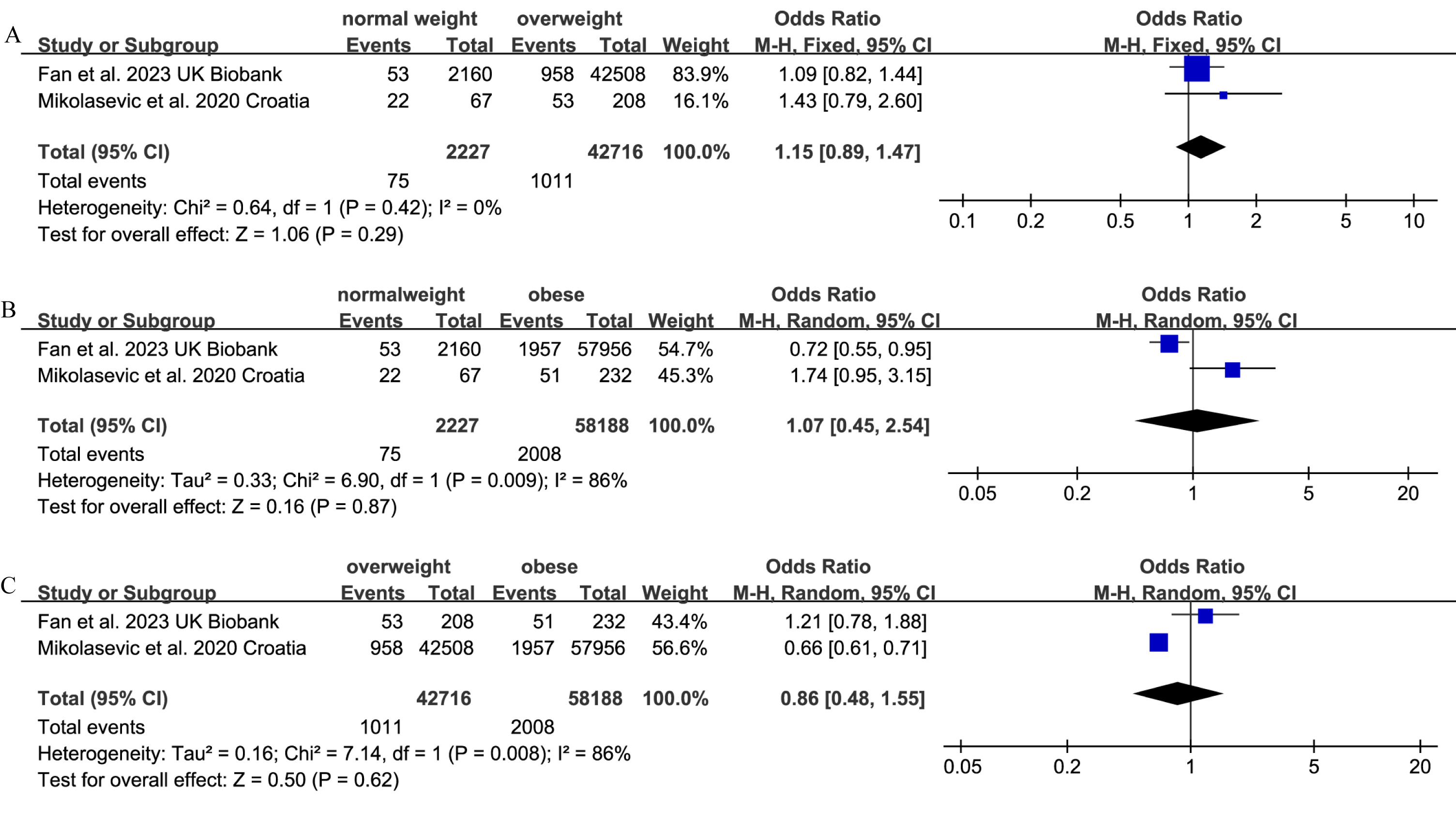

Supplement: Supplemental Information 5 — (A) normal weight versus overweight, (B) normal weight versus obese, (C) overweight versus obese. [file peerj-12-18459-s005.tif]

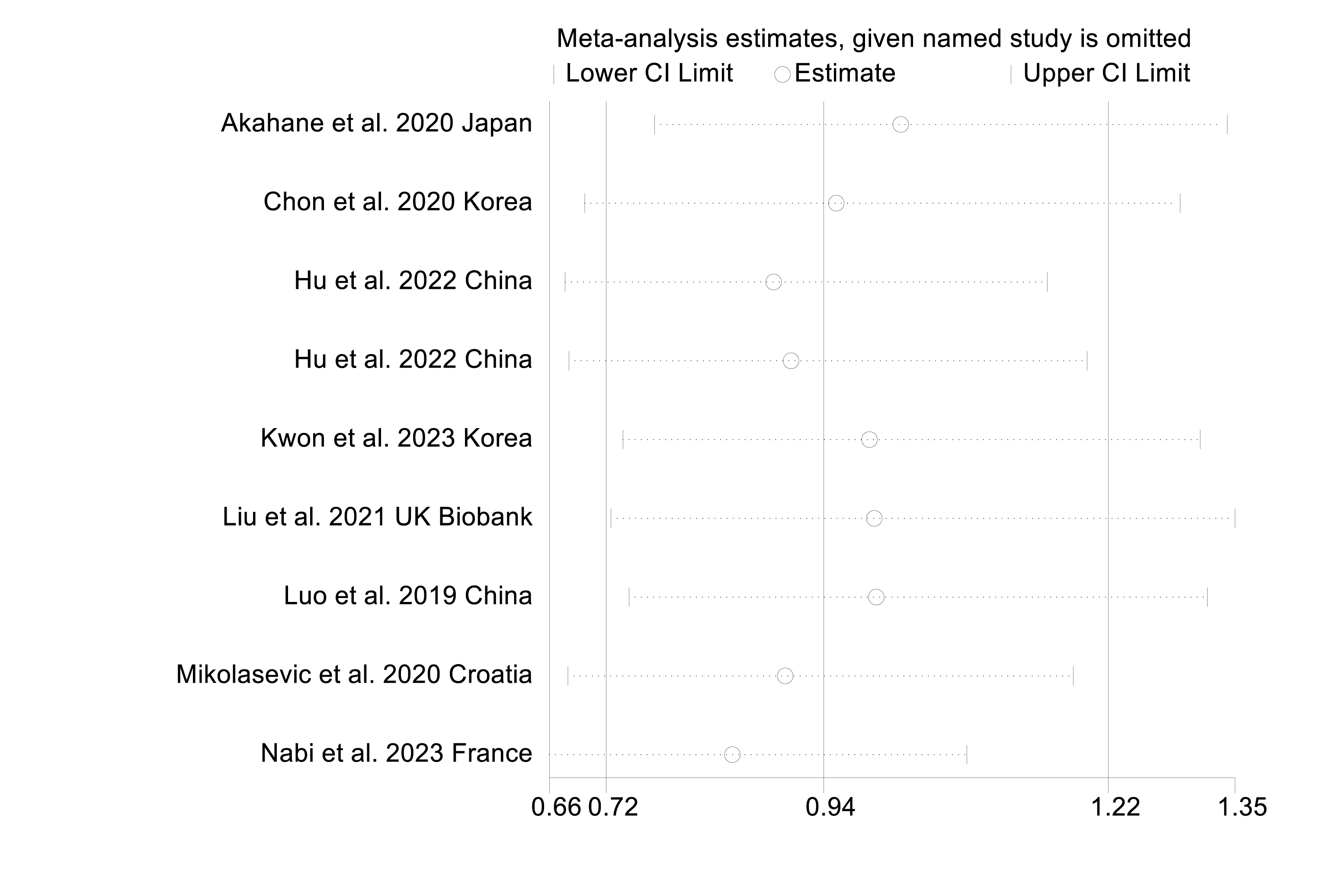

Supplement: Supplemental Information 6 [file peerj-12-18459-s006.tif]

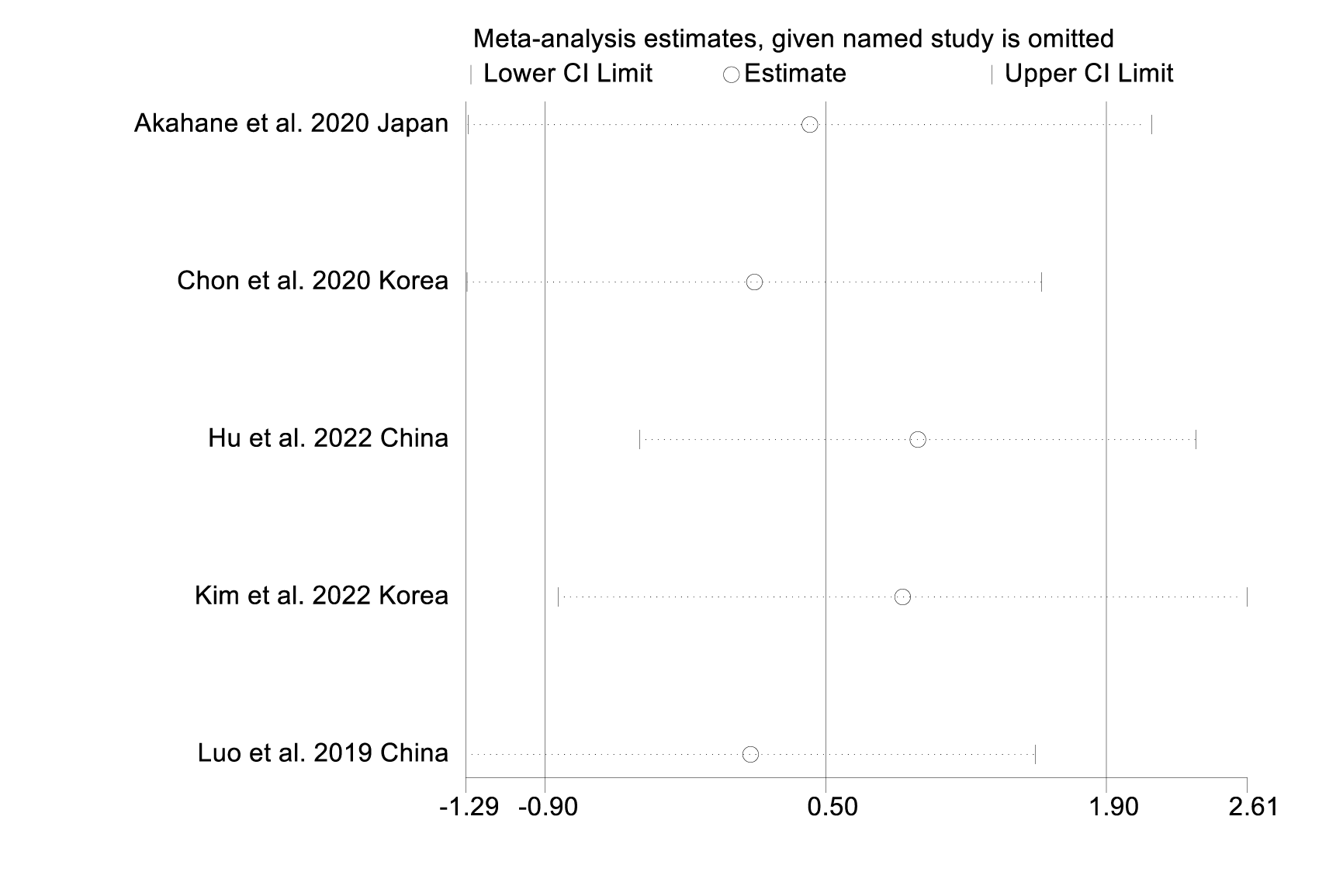

Supplement: Supplemental Information 7 [file peerj-12-18459-s007.tif]

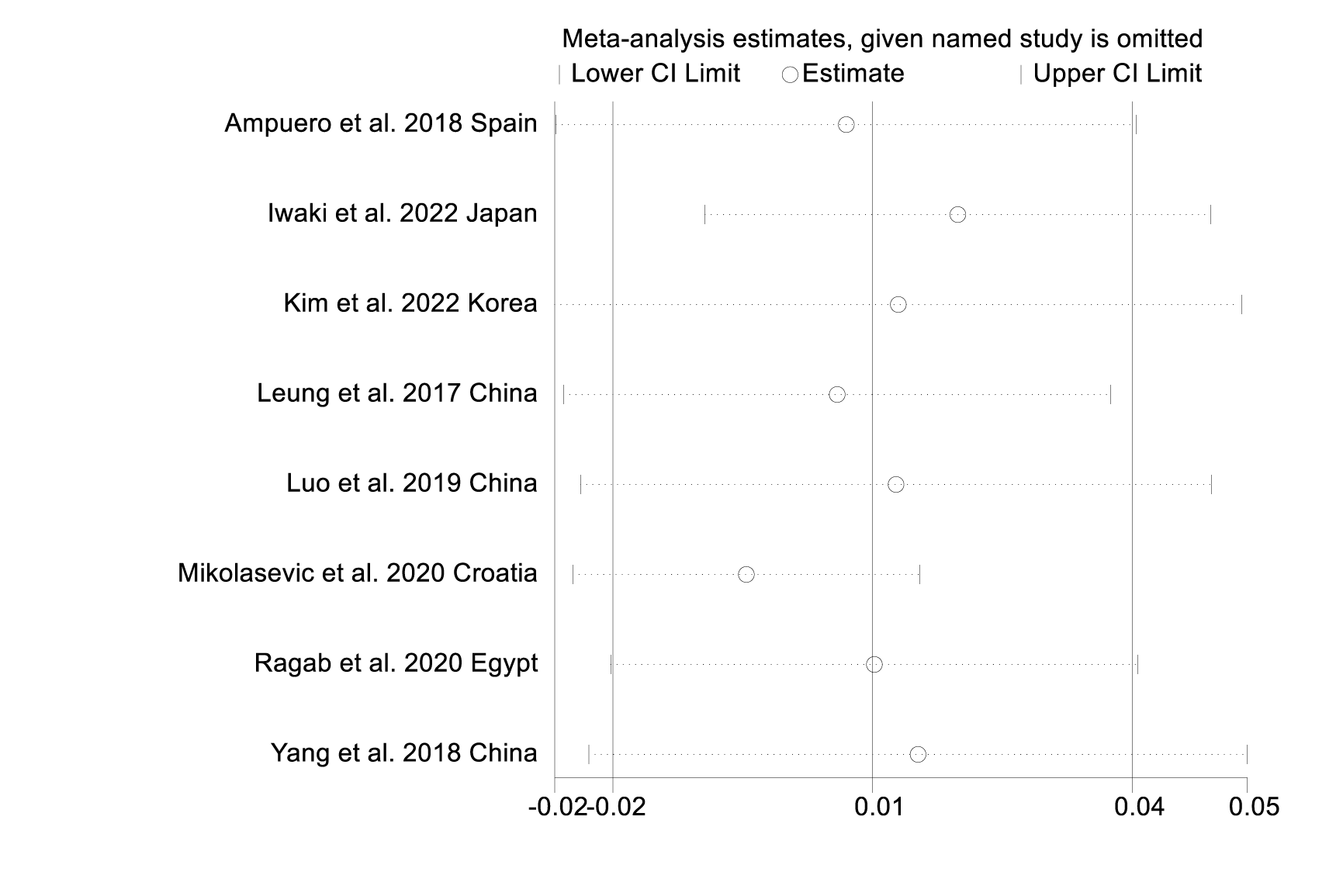

Supplement: Supplemental Information 8 [file peerj-12-18459-s008.tif]
